# Establishing grading indices of available soil potassium on paddy soils in Hubei province, China

**DOI:** 10.1038/s41598-018-33802-3

**Published:** 2018-11-06

**Authors:** Xiaokun Li, Yangyang Zhang, Weini Wang, Muhammad Rizwan Khan, Rihuan Cong, Jianwei Lu

**Affiliations:** College of Resources and Environment, Huazhong Agricultural University; Key Laboratory of Arable Land Conservation (Middle and Lower Reaches of Yangtse River), Ministry of Agriculture; Microelement Research Center, Huazhong Agricultural University, No. 1, Shizishan Street, Hongshan District, Wuhan, 430070 China

## Abstract

Soil testing is an important diagnostic tool for assessing crop-available soil potassium (K) and hence making appropriate fertilizer recommendation. This study was aimed at correlating grain yield response data to soil-test K extracted with ammonium acetate (NH_4_OAc), cold nitric acid (HNO_3_), sodium tetraphenylboron (NaTPB) and boiling HNO_3_ solution, based on 54 field trials conducted during 2011 to 2015 across 15 counties in Hubei province, China. The specific objectives were to establish abundance and deficiency indices of available soil-K (ASK) for rice (*Oryza sativa* L.) and make accurate K fertilizer recommendations. Potassium extracted with NaTPB and boiling HNO_3_ was 1.47 times and 3.61 times higher respectively than that extracted with cold HNO_3_, while K extracted with cold HNO_3_ was 1.32 times higher than that extracted with NH_4_OAc. There were significant logarithmic relationships between crop response and soil-test K. The R^2^ values for cold HNO_3_-K and NaTPB-K methods were much higher than for NH_4_OAc-K method. In order to calibrate the application, the abundance and deficiency indices of ASK categorized by cold HNO_3_-K in low, medium, high and very high ranges were <50 mg kg^−1^, 50 to 100 mg kg^−1^, 100 to 200 mg kg^−1^ and >200 mg kg^−1^ respectively, and that defined by NaTPB-K were <60 mg kg^−1^, 60 to 150 mg kg^−1^, 150 to 330 mg kg^−1^ and >330 mg kg^−1^, respectively. These values could be used to evaluate soil K supplying capacity and make appropriate K fertilizer recommendations for rice.

## Introduction

In agricultural production systems, fertilizer application is required not only to ensure but also to sustain an adequate supply of available nutrients to crops. However, application rates and timing of organic or inorganic fertilizers are often based on an optimal nitrogen supply, but unfortunately, potassium (K) requirement is often neglected. It may lead to an excess or a shortage of K, depending on both crop and soil characteristics^1,2^. A monitoring of soil K reserves is, therefore, extremely important in order to make accurate fertilizer recommendation.

Soil testing is an important diagnostic tool for assessing crop-available soil K in crop production systems. An estimation of exchangeable soil K by extracting air dried soil sample with neutral ammonium acetate (NH_4_OAc) is the most widely used soil-test and provides the basis for K fertilizer recommendations. The inadequacy had been clearly demonstrated in illitic and vermiculitic soil. It does not measure plant-available non-exchangeable K (NEK). Adoption of the Mehlich-3 extractant to estimate plant available K is rapidly increasing in the United States^3^. The Mehlich-3 extractant has been adapted by many laboratories as a near-universal extractant. Soil tests based on these two extractants are suggested for soils of the north-central region by the North-Central Extension and Research Committee for Soil Testing and Plant Analysis, USA^4^. Mengel *et al*. showed that the extraction methods may provide sufficient information for fertilizer recommendations in light textured soils that do not contain 2:1 type clay minerals^5^.

However, the contribution of NEK due to the presence of 2:1 type layer silicates in soils that have the ability to retain K has questioned the validity of using exchangeable K soil extraction methods. In some cases, the non-exchangeable K pool (i.e. K in interlayer sites) can make a considerable contribution (80–100%) to plant available K^6^. The role of this pool becomes particularly important under K-mining conditions when the exchangeable K is low. The significance of the contribution of NEK has probably been underestimated. Nevertheless, various methods have been established to assess slowly or potentially available K in soils, e.g. extraction with hydrochloric acid (HCl), boiling in nitric acid (HNO_3_), electro-ultrafiltration, exchange resins, sodium tetraphenylboron (NaTPB), and field balances^7^.

It was found that Step-K (loosely bound NEK released by repeated extraction with HNO_3_) was a more reliable index of the K-supplying power of Canadian soils than is NH_4_OAc-extractable K. However, this approach is not suited for routine soil test purposes because of the extra equipment, bench space, and time required to estimate K. Soil K extraction with NaTPB is characterized by the formation of potassium tetraphenylboron (KBPh_4_) precipitate, which reduces the concentration of K^+^ in soil solution, thus facilitating the further release of K^+^ from the interlayer sites of soil minerals^8^. The NaTPB method is not only used for characterizing the release of NEK from soil or minerals, but has also been shown to be a good method for determining soil K availability to plants^9,10^. This method was first proposed by Hanway in 1956 and since then gradually modified by many researchers^9,11^. The 60-min modified NaTPB method without sodium chloride (0.2 mol L^−1^ NaTPB) is suitable for evaluating K availability to plants in a variety of soils^12^.

In order to provide theoretical basis for rice production and fertilization by soil testing, 54 field experiments regarding rice yield response to K application were carried out at different locations during 2011–2015. Available K in soils were evaluated using different extractants, i.e. 1.0 mol L^−1^ neutral NH_4_OAc, 2.0 mol L^−1^ cold HNO_3_, 1.0 mol L^−1^ boiling HNO_3_ and 0.2 mol L^−1^ NaTPB, respectively. The specific objectives were (1) to establish abundance and deficiency indices of ASK for rice, (2) to find the most suitable method for assessing ASK and making accurate K fertilizer recommendations.

## Results

### Amount of soil K extracted

**Figure 1:**
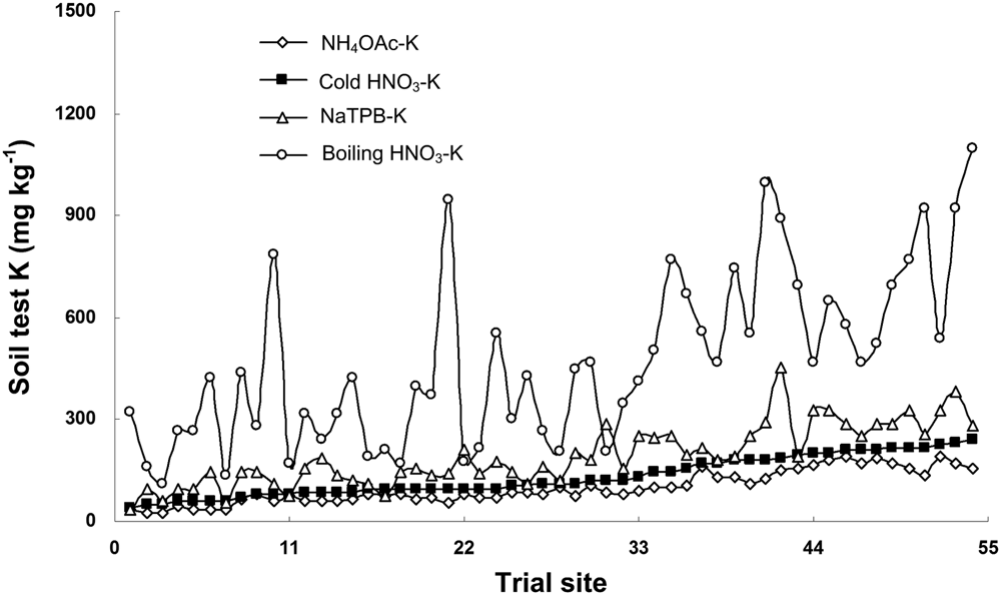
Concentrations of soil-test K extracted with 1.0 mol L^−1^ NH_4_OAc, 2.0 mol L^−1^ cold HNO_3_, 0.2 mol L^−1^ NaTPB and 1.0 mol L^−1^ boiling HNO_3_ solution across 54 trial sites. Trial sites were sequenced by the value of soil cold HNO_3_-K from small to large.

The concentrations of cold HNO_3_-K, NH_4_OAc-K, NaTPB-K and boiling HNO_3_-K of non-fertilized soil across all site-years ranged from 40.0 to 242.4 mg kg^−1^, 22.9 to 192.6 mg kg^−1^, 36.1 to 451.6 mg kg^−1^ and 109.8 to 1095.3 mg kg^−1^ respectively, average values being 130.5 mg kg^−1^, 98.6 mg kg^−1^, 192.1 mg kg^−1^ and 471.0 mg kg^−1^ respectively (Fig. 1). It showed that the mean values of NaTPB-K and boiling HNO_3_-K were 1.47 times and 3.61 times higher than of cold HNO_3_-K. Similarly, the mean values of cold HNO_3_-K was 1.32 times higher than that of NH_4_OAc-K.

Table 1 showed relationships between soil test K extracted with 1.0 mol L^−1^ NH_4_OAc, 2.0 mol L^−1^ cold HNO_3_, 0.2 mol L^−1^ NaTPB-EDTA and 1.0 mol L^−1^ boiling HNO_3_ solution. There were significant correlations between soil-test K extracted with different solutions. The strength of the relationship was very strong for cold HNO_3_-K and NH_4_OAc-K, and for NaTPB-K and boiling HNO_3_-K (*r* 0.957 and 0.848, respectively).

**Table 1:** Correlation between soil-test K extracted with 1.0 mol L^−1^ NH_4_OAc, 2.0 mol L^−1^ cold HNO_3_, 0.2 mol L^−1^ NaTPB and 1.0 mol L^−1^ boiling HNO_3_ solution.

	NH_4_OAc-K	Cold HNO_3_-K	NaTPB-K	Boiling HNO_3_-K
NH_4_OAc-K	—	—	—	—
Cold HNO_3_-K	0.957**	—	—	—
NaTPB-K	0.817**	0.848**	—	—
Boiling HNO_3_-K	0.584**	0.678**	0.639**	—

**Refers to significant correlation at *P* < 0.01.

### Grain yield and K uptakes response to K application

**Figure 2:**
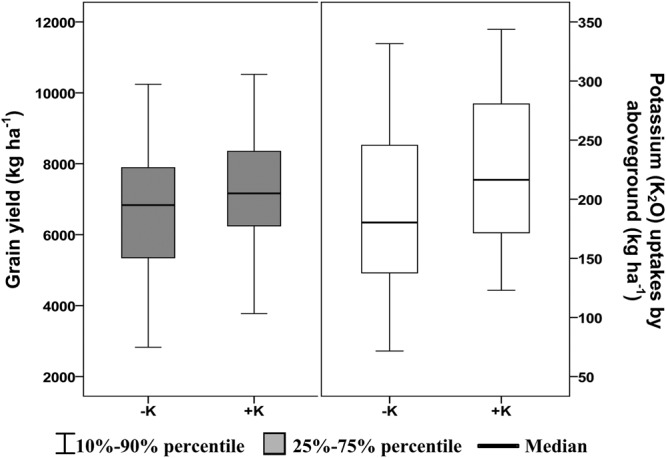
Rice grain yield and K uptake by aboveground for all trails sites.

Regression equations were established to investigate the influences of K fertilizer levels on grain yields and hence calculate the optimum amount of fertilizer required to obtain the highest yield at each site (data did not shown). The wide range of growing conditions considered in this study resulted in grain yields varying from 2825 to 10240 kg ha^−1^ in non-fertilized plots and from 3775 to 10520 kg ha^−1^ in optimum K fertilized plots (Fig. 2). Analysis of variance of K effects on yield for individual sites (not shown) indicated a significant yield increase (*P* < 0.05), on average 10% increase due to K fertilization of the rice site-years. Potassium uptake by aboveground parts ranged from 71.6 to 331.6 kg ha^−1^ in non K fertilized plots and from 122.9 to 343.7 kg ha^−1^ in K fertilized plots. It varied across different sites. Potassium fertilizer application significantly improved K uptakes (*P* < 0.05). On average, K uptake in response to K fertilization was increased by 18.9% significantly (*P* < 0.05).

**Figure 3:**
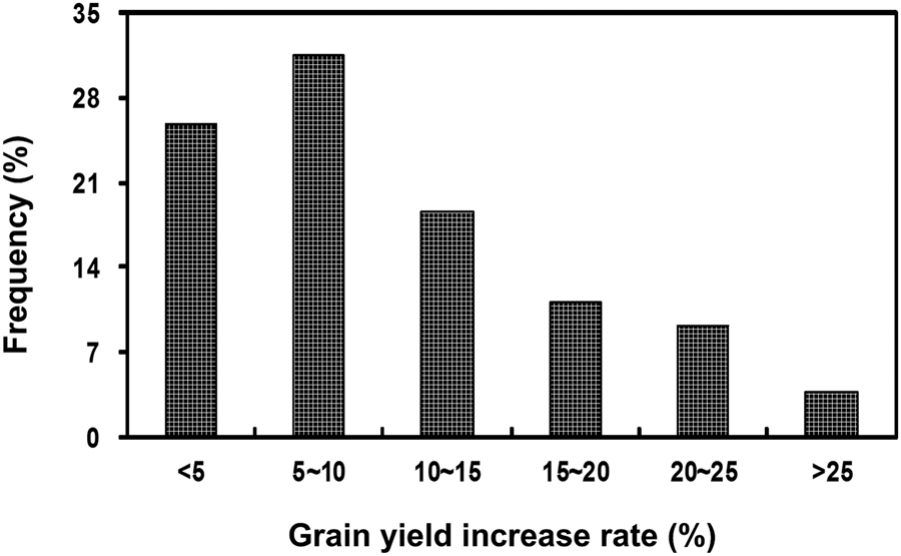
Frequency distribution of grain yield increase rate of the + K treatment compared to –K treatment for the 54 trails.

The distribution of grain yield increase rate due to K fertilization in 54 trails was illustrated in Fig. 3. It showed a significant unimodal distribution, with 5%~10% as the crest. Grain yield of 25.9% of the 54 trials did not respond significantly to K fertilization (increase rate <5%). The percentage of the 54 trials with grain yield increase rate at 5%~10%, 11%~15%, 16%~20%, 21%~25% and >25% were 31.5%, 18.5%, 11.1%, 9.3% and 3.7%, respectively. It indicated that soil K status of the rice trials was uniformly distributed, which was suitable to establish the abundance and deficiency indices of ASK for rice.

### Relationship between soil-test K and crop yield response to K

**Figure 4:**
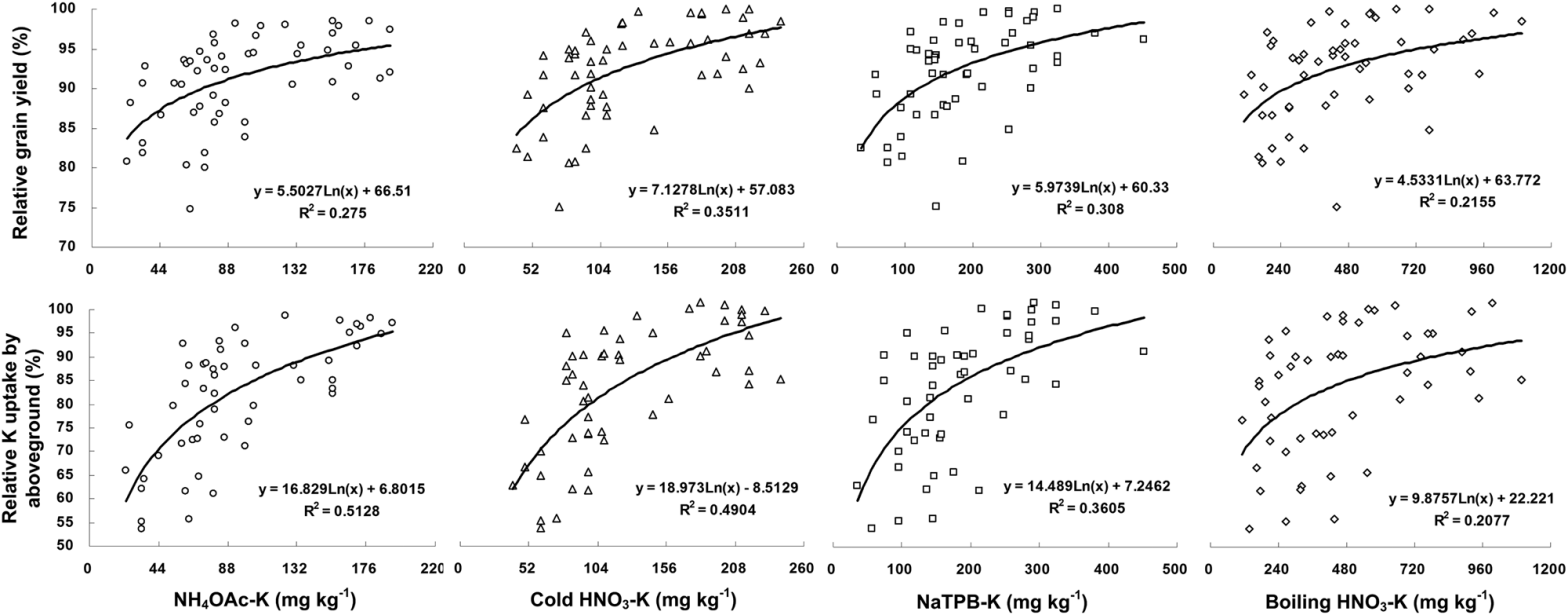
Relationships between K extracted from non-fertilized samples and rice relative grain yield, relative K uptakes response to K fertilization. Lines represent the fit of a logarithm model.

Figure 4 showed relationship between relative grain yield (RGY), relative K uptake (RKU) and soil text K extracted with different extractants. The data showed the known general relationship between crop yield response and soil-test values, but also showed high unexplained variability. The R^2^ values ranged from 0.2155 to 0.3511 for RGY and ranged from 0.2077 to 0.5128 for RKU. There were small differences between soil-test methods. It also indicated high efficiency of cold HNO_3_-K and NaTPB-K methods to assess crop K sufficiency and predict crop response to K fertilization in rice.

Table 2 showed abundance and deficiency indices of ASK for rice calculated by the logarithmic models. Abundance and deficiency indices of ASK based on regression equation greatly varied depending on the soil-test method and model used. In four methods, the abundance and deficiency indices values were lowest with NH_4_OAc-K method, intermediate with cold HNO_3_-K and NaTPB-K method, and highest with boiling HNO_3_-K method. In order to facilitate the application, the adjusted abundance and deficiency indices of ASK for low, medium, high and very high ranges defined by cold HNO_3_-K were <50 mg kg^−1^, 50 to 100 mg kg^−1^, 100 to 200 mg kg^−1^ and >200 mg kg^−1^, respectively. The corresponding values defined by NaTPB-K were <60 mg kg^−1^, 60 to 150 mg kg^−1^, 150 to 330 mg kg^−1^ and >330 mg kg^−1^, respectively. According to established abundance and deficiency indices of ASK, average cold HNO_3_-K values for low, medium, high and very high levels in this study were 45.7, 83.6, 150.4 and 218.1 mg kg^−1^ respectively, and the average grain yield increase rates were 19.9%, 13.6%, 7.4% and 5.7% respectively. Similarly, average NaTPB-K values for low, medium, high and very high levels were 50.8, 119.9, 236.6 and 416.4 mg kg^−1^ respectively, and the average grain yield increase rates were 15.4%, 13.8%, 7.9% and 5.2% respectively. It demonstrated that abundance and deficiency indices of ASK could help evaluate soil K supplying capacity and provide evidence for making accurate K fertilizer recommendations.

**Table 2:** Abundance and deficiency indices of available soil potassium (ASK) extracted with 1.0 mol L^−1^ NH_4_OAc, 2.0 mol L^−1^ cold HNO_3_, 0.2 mol L^−1^ NaTPB and 1.0 mol L^−1^ boiling HNO_3_ solution for rice.

	NH_4_OAc-K				Cold HNO_3_-K				NaTPB-K				Boiling HNO_3_^−^K			
	Low	Medium	High	Very high	Low	Medium	High	Very high	Low	Medium	High	Very high	Low	Medium	High	Very high
Relative grain yield (%)	<85	85~90	90~95	>95	<85	85~90	90~95	>95	<85	85~90	90~95	>95	<85	85~90	90~95	>95
ASK based on regression equation (mg kg^−1^)	<29	29~71	71~177	>177	<50	50~101	101~204	>204	<62	62~144	144~331	>331	<108	108~326	326~981	>981
Adjusted ASK (mg kg^−1^)	<30	30~70	70~180	>180	<50	50~100	100~200	>200	<60	60~150	150~330	>330	<110	110~330	330~980	>980
Percentage of the total number of samples (%)	3.8	28.3	62.3	5.7	5.7	39.6	35.8	18.9	5.7	35.8	54.7	3.8	1.9	35.8	58.5	3.8
Mean (mg kg^−1^)	24.1	53.6	112.9	190.4	45.7	83.6	150.4	218.1	50.8	119.9	236.6	416.4	109.8	233.3	591.5	1045.0
Average grain yield increase rate (%)	18.7	14.3	8.4	5.1	19.9	13.6	7.4	5.7	15.4	13.8	7.9	5.2	13.4	14.5	8.2	2.6

## Discussion

For soil-test-based K fertilizer recommendations to be accurate, the relationship between the soil K availability index and rice yield must be determined and followed by calibrating the proper K fertilizer rates to the soil nutrient availability index^13^. This research found that there were significant logarithmic relationships between rice yield response and soil-test K extracted with NH_4_OAc, cold HNO_3_, NaTPB and boiling HNO_3_. The cold HNO_3_-K and NaTPB-K were relatively more efficient in predicting rice response to K fertilization compared with NH_4_OAc-K. It indicated that cold HNO_3_-K and NaTPB-K methods should be appropriate for rice in Hubei Province, China. However, Slaton *et al*. correlated relative rice yield with Mehlich-3 and 1 mol L^−1^ HNO_3_ extractable K and suggested Mehlich-3 soil and plant K critical concentrations should be appropriate for U.S. mid-South rice-producing areas^13^. This showed that the method for evaluating soil available K for rice differed in regions. Adoption of the Mehlich-3 extractant to estimate plant available K, which includes NH_4_OAc in its extracting solution^3^, is increasing rapidly in the United States. However, Nair *et al*. showed that NH_4_OAc and Mehlich-3 extractable K lone was a poor indicator of K availability to cardamom in kaolinitic soils, unless it is integrated with soil K buffer power^14^. It indicated that the NH_4_OAc and Mehlich-3 methods were affected by soil clay mineral composition. Potassium release from soil is dependent on the type and amount of K-bearing minerals^15^. Fixation and release of NEK affects the abundance and binding strength of exchangeable K, as well as quantity-intensity factors^16^. There are two ways to solve this problem. One is that we establishing grading indices of available soil potassium according to soil type, the other one is that we find a uniform method to evaluate plant-available K in a variety of soils.

In general, soil K is understood to exist in four distinct K pools that differ in their accessibility to plant roots, with reversible transfer of K between the pools^17^. The water soluble and exchangeable forms are regarded as rapidly available forms of K: they are replenished by NEK when they are depleted as a result of plant removal and/or leaching^18^. Wang *et al*. reported that the amounts of maximum NEK accounted for 21–56% of the total K of the soils tested^19^. The plant availability of NEK depends primarily on the rate at which it can be released as more labile forms (i.e., both exchangeable and soluble)^20^. Some researchers suggest that the more rapidly NEK is released, the more easily it is utilized by plants^21^. Li *et al*. demonstrated that NEK was the main form of K available to rice, followed by exchangeable K and water soluble K in the yellow cinnamon soil in Hubei province^22^. NaTPB-K was a good methods for grading NEK bioavailability. Plant-available K in soils was classified into three categories: high available K, medium available K and low available K, and grading criteria and measurement methods were also proposed^23^. Better relationship of relative yield and NaTPB-K compared with other methods (Fig. 4) showed that soil available K extracted with NaTPB could be appropriate for paddy soils in Hubei Province, China. The abundance and deficiency indices of ASK as defined by NaTPB-K (Table 4) should be used to make K fertilizer recommendations for rice in this region. Traditionally, K availability in soils is studied by its extraction with neutral 1.0 mol L^−1^ NH_4_OAc. However, cereal crops like rice, wheat and ryegrass etc. have strong K absorption capacity^24,25^. Many experiments proved that non-exchangeable K was the main source of K in this type of crops^26,27^. Obviously, it is not enough just using exchangeable K evaluating soil K supplying capacity for rice. Bao and Shi proposed using cold HNO_3_-K as the method of determining the available K of paddy soil^28^. This paper showed that soil available K extracted with cold HNO_3_ could also be appropriate for paddy soils in Hubei Province, China, compared with that of NH_4_OAc-K (Fig. 4). So, respective grading indices of available soil K on paddy soils were established (Table 4). It can be selected based on actual experimental conditions.

## Conclusions

Grain yield and K uptake of rice in Hubei province, China, in response to K fertilization which varied across different sites were increased significantly, on average, by 10.0% and 18.9%, respectively. The grain yield increase rate exhibited a significant unimodal distribution, with 5%~10% as the crest. There were significant logarithmic relationships between crop yield response and soil-test K. The cold HNO_3_-K and NaTPB-K were relatively more efficient in predicting rice response to K fertilization compared with NH_4_OAc-K. The abundance and deficiency indices of ASK for low, medium, high and very high ranges as defined by cold HNO_3_-K were <50 mg kg^−1^, 50 to 100 mg kg^−1^, 100 to 200 mg kg^−1^ and >200 mg kg^−1^ respectively, and that defined by NaTPB-K were <60 mg kg^−1^, 60 to 150 mg kg^−1^, 150 to 330 mg kg^−1^ and >330 mg kg^−1^, respectively. It could be used to evaluate soil K supplying capacity and hence making K fertilizer recommendations for rice in Hubei province, China.

## Materials and Methods

**Figure 5:**
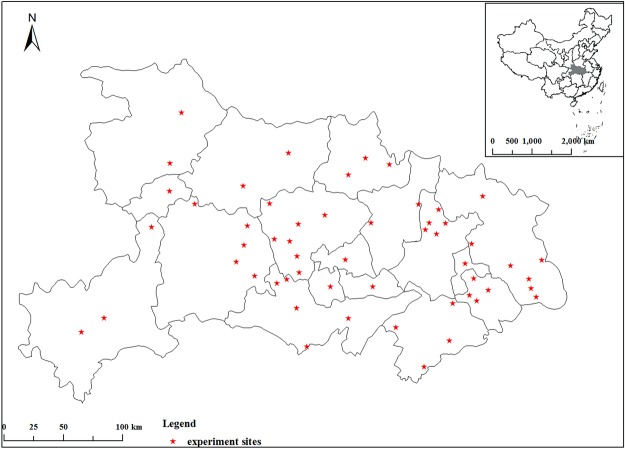
Location of experiment field site.

Rice grain yield data and soil samples were collected from K response trials conducted during 2011 to 2015 across 15 counties in Hubei province, China where oilseed rape (*Brassica napus* L.) - rice rotation system predominates (Table 3). A total of random 54 field trials of K application response were conducted in farmers’ fields which belong to the main rice producing areas (Fig. 5). Each trial was comprised of four K fertilizer (granulated potassium chloride, 500 g K kg^−1^) treatments (K_0_, K_1_, K_2_ and K_3_) applied as basal fertilizer. Nitrogen (N) and phosphorus (P) fertilizers were applied across all plots of each trial at rates that were recommended by local Agricultural Technology Promotion Center and Huazhong Agricultural University (Table 4).

**Table 3:** Selected properties of the 54 soils included in the trials.

No.	pH	Organic matter (g kg^−1^)	Total N (g kg^−1^)	Avail. P (mg kg^−1^)	NH_4_OAc K (mg kg^−1^)	No.	pH	Organic matter (g kg^−1^)	Total N (g kg^−1^)	Avail. P (mg kg^−1^)	NH_4_OAc K (mg kg^−1^)
1	6.08	38.40	2.11	18.9	47.1	28	7.86	55.23	2.76	23.7	109.4
2	6.68	28.15	1.91	10.9	82.8	29	6.23	23.03	1.52	17.8	170.5
3	6.75	32.15	1.46	12.0	44.5	30	7.96	38.50	2.22	27.2	155.2
4	5.57	23.82	1.25	9.6	33.1	31	6.45	20.67	1.17	42.5	155.2
5	5.76	27.18	1.61	7.1	63.6	32	6.60	44.93	2.64	21.3	134.9
6	6.86	22.19	1.38	7.7	58.5	33	6.76	44.37	2.36	46.2	124.7
7	5.64	30.32	1.54	10.6	22.9	34	5.26	30.50	1.68	15.5	155.2
8	6.90	26.83	1.69	18.8	101.2	35	7.99	14.26	1.20	12.5	104.3
9	6.61	31.98	1.44	10.4	61.3	36	6.8	23.2	1.30	5.6	106.3
10	6.05	33.06	1.55	6.9	65.7	37	5.8	22.5	1.40	9.4	152.8
11	5.92	35.26	1.89	25.9	61.3	38	5.0	20.7	1.30	12.5	79.7
12	5.70	30.60	2.00	7.9	34.8	39	6.6	24.2	1.30	10.0	59.8
13	5.90	31.07	1.74	12.8	68.7	40	5.3	18.0	1.00	5.0	86.3
14	5.63	30.14	1.94	18.9	63.6	41	4.6	23.2	0.80	5.2	86.3
15	6.07	38.71	2.06	11.4	170.5	42	5.7	26.4	1.40	3.1	166.0
16	5.81	24.61	1.45	8.7	99.2	43	7.1	26.7	1.50	12.5	179.3
17	6.68	10.11	0.61	7.5	129.8	44	7.7	30.2	1.80	3.5	93.0
18	5.43	25.70	1.24	43.3	84.0	45	5.6	26.5	1.80	30.2	79.7
19	5.28	22.42	1.26	8.4	78.9	46	6.0	26.9	0.61	8.1	192.6
20	5.00	19.90	1.25	60.2	99.2	47	5.3	27.3	0.70	17.4	159.4
21	7.24	18.35	1.11	10.3	53.4	48	5.18	30.12	1.54	12.73	73.1
22	5.73	21.59	1.26	15.1	33.1	49	4.98	33.02	1.67	12.16	73.1
23	5.95	16.88	1.10	10.5	78.9	50	4.97	32.80	1.6	14.3	79.7
24	5.24	23.47	1.27	35.6	70.3	51	4.76	25.6	2.00	2.32	33.2
25	5.68	22.52	1.17	10.9	74.5	52	6.80	21.3	1.16	12.62	192.6
26	5.96	26.48	1.49	7.1	70.0	53	6.20	30.5	1.10	12.81	132.8
27	5.64	23.06	1.62	20.8	25.4	54	6.00	29.9	1.31	10.68	172.7

Avail., available.

**Table 4:** Range and average of fertilizer application rate of the 54 trials.

Treatment	N		P_2_O_5_		K_2_O	
	Range	Ave.	Range	Ave.	Range	Ave.
K_0_	135~180	166 ± 12	45~90	62 ± 13	0	0
K_1_	135~180	166 ± 12	45~90	62 ± 13	22~60	41 ± 24
K_2_	135~180	166 ± 12	45~90	62 ± 13	45~120	85 ± 29
K_3_	135~180	166 ± 12	45~90	62 ± 13	67~180	128 ± 34

Rice was manually transplanted in all the trials. Other management practices such as rice cultivars, seedling rates, transplanting dates and weed control measures were those normally used at each county. At physiological maturity, rice grain yield was determined from an area of 10 m^2^ in each plot through manual harvesting and mechanical threshing and adjusted to the standard moisture content of 14%; Plant samples were collected from 6 ridges per plot and separated into straw and grain. Total K content of plant material was determined after dry ashing method in a muffle furnace at 500 °C for 2 h^29^.

Composite soil samples (12–15 cores) were collected from 0 to 20 cm soil depth before applying K fertilizer. Samples were air dried and were crushed to pass through a 0.84 mm sieve to determine soil K content. Four forms of soil K have been recognized, and they are structural or mineral K, non-exchangeable K (NEK), exchangeable K and water-soluble K. Exchangeable K and water-soluble K are often considered readily available to plants, while NEK and Min-K are only slowly or potentially available. Because soil NEK may become available to crops, the soil available K can be determined by the amount of exchangeable K and NEK in soils. NH_4_OAc-K was determined by extracting 2.5 g of soil with 25 mL neutral 1.0 mol L^−1^ NH_4_OAc for 30 min and filtering through filter paper. Cold HNO_3_-K was determined by extracting 2.5 g of soil with 50 mL cold 2 mol L^−1^ HNO_3_ for 30 min and filtering through filter paper. NaTPB-K was extracted using the mixture of NaTPB and ethylenediaminetetraacetic acid (EDTA). Samples of 0.5 g of soil were weighed into 50-mL centrifuge tubes and then 3 mL of extractant (0.2 mol L^−1^ NaTPB_ + _0.01 mol L^−1^ EDTA) was added. After shaking at 200 r.p.m. for 60 min, 25 mL of quenching solution (0.5 mol L^−1^ NH_4_Cl + 0.14 mol L^−1^ CuCl_2_) was poured into the tubes to stop the extraction of soil K. The tubes were then heated in boiling water for 60 min to dissolve the KBPh_4_ precipitate. The supernatant was separated by centrifugation for 10 min and was used later for K determination after dilution. Similarly, boiling HNO_3_-K was determined by extracting 2.5 g of soil with 25 mL 1.0 mol L^−1^ boiling HNO_3_ for 10 min and filtering through filter paper. The concentration of K in plant and soil samples was determined using flame photometer.

Relative grain yield (RGY) was calculated using equation (1).

1 $$\mathrm{RGY}( \% )=\frac{{\rm{Grain}}\,{\rm{yield}}\,{\rm{of}}\,{\rm{treatment}}\,{\rm{without}}\,{\rm{K}}\,{\rm{fertlizer}}}{{\rm{Grain}}\,{\rm{yield}}\,{\rm{of}}\,{\rm{treatment}}\,{\rm{with}}\,{\rm{K}}\,{\rm{fertilizer}}}\times {\rm{100}}$$

Relative K uptake by crops (RKU) was calculated using equation (2).

2 $${\rm{RKU}}( \% )=\frac{{\rm{K}}\,{\rm{uptake}}\,{\rm{by}}\,{\rm{crops}}\,{\rm{of}}\,{\rm{treatment}}\,{\rm{without}}\,{\rm{K}}\,{\rm{fertilizer}}}{{\rm{K}}\,{\rm{uptake}}\,{\rm{by}}\,{\rm{crops}}\,{\rm{of}}\,{\rm{treatment}}\,{\rm{with}}\,{\rm{K}}\,{\rm{fertilizer}}}\times 100$$

Pairs of matching RGY and corresponding soil-test K values for each site-year are represented by one point in figures. The abundance and deficiency indices of soil-test K for rice were calculated using the logarithmic equation models^30^ and the statistical Cate-Nelson method^31^. According to the method of the reference and the actual situation of this study, soil-test K was categorized as low, medium, high and very high based on the corresponding relative yield values which were <85%, 85~90%, 90~95% and >95%, respectively.
